# Reversal of precedence: The oldest available name of the Javan gibbon and a complete synonymy of the species

**DOI:** 10.1007/s10329-020-00822-5

**Published:** 2020-04-24

**Authors:** Kai R. Caspar

**Affiliations:** Department of General Zoology, Faculty of Biology, University of Duisburg-Essen, Essen, Germany

**Keywords:** Nomenclature, Reversal of precedence, *Hylobates moloch*, Synonymy, Nomen oblitum, History of primatology

## Abstract

Over the course of their long research history, a plethora of scientific names have been applied to the different species of apes (Hominoidea). Although numerous authors, past and present, have attempted to reconstruct hominoid nomenclatural history in detail, it appears that parts of it, mostly concerning the lesser apes or gibbons, still remain overlooked. Here I show that *Simia Nanodes* Lichtenstein, 1791 is a senior synonym of *Simia Moloch* Audebert, 1797, otherwise regarded as the oldest binomen applicable to the Javan gibbon. However, since *Simia Nanodes* failed to enter common usage, these names are subject to a reversal of precedence as defined by Article 23.9 of the International Code of Zoological Nomenclature. *Simia Moloch* Audebert, 1797 must be maintained as a *nomen protectum*, resulting in *Hylobates moloch* (Audebert, 1797) staying the valid name of the species*. Simia Nanodes* Lichtenstein, 1791 is declared a *nomen oblitum*. In compliance with this, I provide a complete scientific synonymy of the Javan gibbon and comment on its early research history.

**Electronic supplementary material:**

The online version of this article (10.1007/s10329-020-00822-5) contains supplementary material, which is available to authorized users.

The history of primate research and nomenclature is the subject of ongoing scientific discussion, with that of apes (Hominoidea) being of particular concern. In the case of great apes (Hominidae) this interest continues to regularly spawn new monographs (e.g., Ingensiep 2013; Herzfeld 2017), at times still resulting in nomenclatural revisions (Oates et al. 2009). Compared to that, the lesser apes or gibbons (Hylobatidae) appear to be covered only superficially. In recent times, their research history, including nomenclature, was most importantly discussed and analyzed by Groves (1972, 2008) but otherwise received only poor attention.

The Javan or silvery gibbon, *Hylobates moloch* (Audebert 1797) (Fig. 1a), is a well-studied species of lesser ape, whose research history spans several centuries. It is exclusively found on Java and is the only extant hylobatid inhabiting the island. For decades, *Simia Moloch* Audebert, 1797 has been universally accepted as the oldest available name for this species, as established by Cabrera (1930). However, mentions of Javan gibbons significantly predate the description by Audebert (1797). The earliest reference at times attributed to the Javan gibbon dates to the latest 17th century (Le Comte 1696) but its ambiguous geographical and morphological descriptions make it impossible to confidently assign this report to one particular species of lesser ape. The first unequivocal references to Javan gibbons were only provided several decades later. Pennant (1771) mentioned a gibbon in the possession of Lord Clive that was “good-natured, and full of frolick”. It was described as having a silvery pelage with a black cap and therefore surely was a Javan gibbon. More detailed reports on the species were provided by contemporary Dutch scholars, while Java was controlled by the Dutch East India company. Most notably, Van Iperen and Schouwman (1780) but also Camper (1779, 1782) wrote about its natural history and anatomy. Van Iperen and Schouwman (1780) were the only European scholars of the 18th century that gave detailed first-hand accounts of living Javan gibbons, which they observed in captive settings while staying on the island. They not only provided accurate descriptions of their appearance but also reported on behavioral aspects and folkloristic knowledge about the species. Furthermore, they presented a list of anatomical measurements from two adult Javan gibbons. By the early 1780s, multiple specimens had been sent to Europe. Some were housed in the Dutch Stadholder’s collection in the Hague and in the Museum Leverianum in London (Camper 1782). An adult female specimen in the Leverian collection stood model for what might be the oldest surviving illustration of the species (Shaw 1792; Fig. 1b, later redrawn by Pennant (1793) whose depiction was copied by Schreber (1799)). Van Iperen and Schouwman (1780) suggested that the Javan gibbon represented a different species from the “gibbon of Buffon” (now *Hylobates lar* (Linnaeus, 1771), the white-handed or lar gibbon), the first small ape known to science (Buffon 1766), but refrained from providing a binomen for it. Neither did Camper (1782), who unified the two. Instead, these authors used the name *Wouwouw* or *Wou-wou* when referring to the ape, an onomatopoeic expression alluding to its characteristic vocalizations. In the late 18th and early 19th centuries, this trivial name was applied only to the Javan gibbon, but some scholars later extended it to the agile gibbon, *Hylobates agilis* F. Cuvier, 1821 (Martin 1841).

**Fig. 1:**
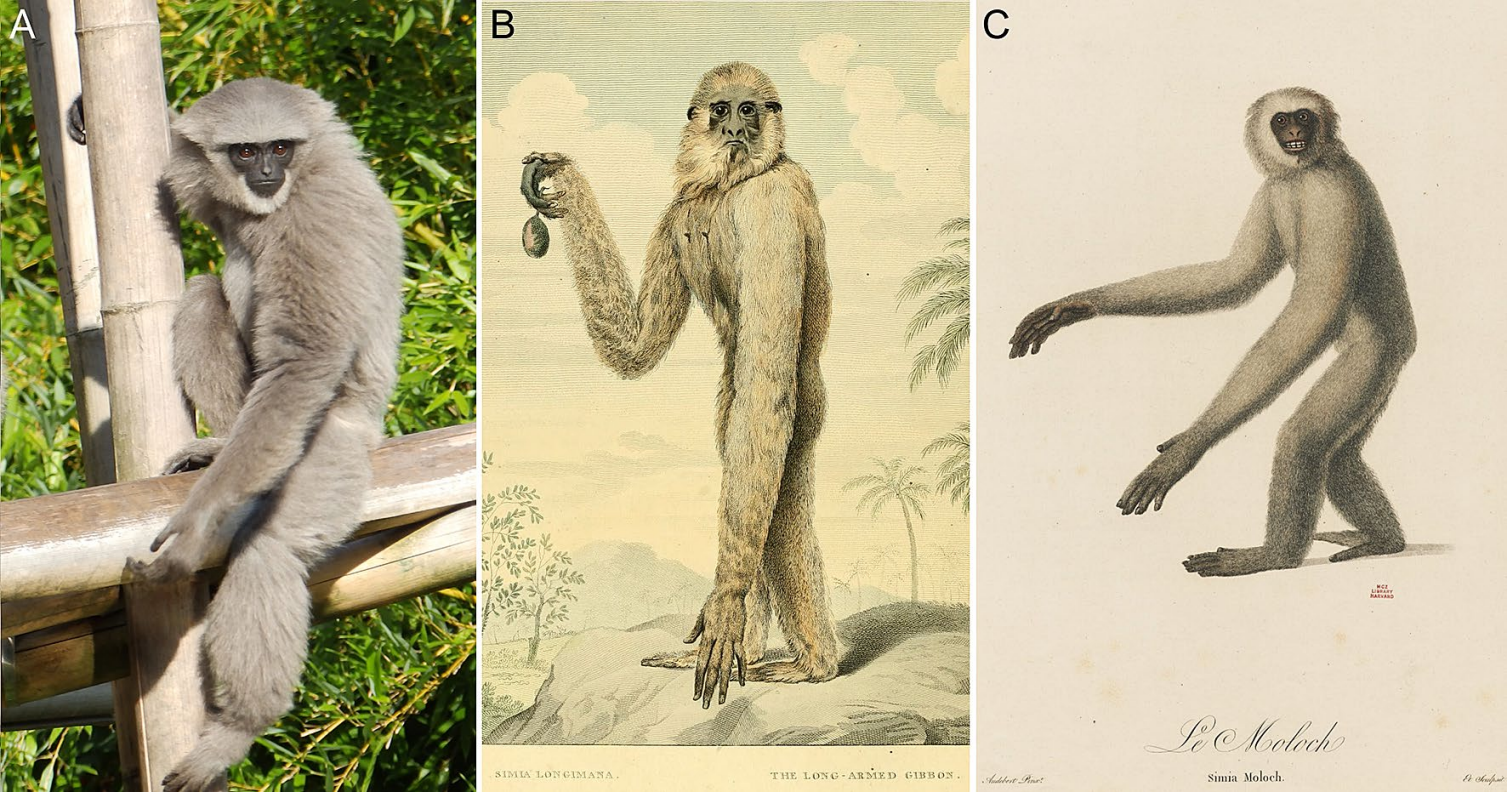
The Javan gibbon and early scientific illustrations of it. **a** Captive subadult Javan gibbon. Photograph by K. R. Caspar. **b** Possibly the oldest surviving artistic depiction of the species by Western authors from Shaw (1792) (Van Iperen and Schouwman (1780) hint at older ones drawn from live in Java), showing a stuffed female specimen from the since disintegrated Leverian collection in London in an imaginative landscape. The animal is wrongly identified as a white-handed gibbon (= *Simia longimana*) in the picture caption. **c** Type illustration of *Simia Moloch* from Audebert (1797) based on the lectotype of the Javan gibbon preserved at the Muséum National d’Histoire Naturelle in Paris (MNHN-ZM-2005-970). All individuals depicted only show traces of the black cap that is typically expressed in the species. The missing caps in the oldest illustrations of Javan gibbon specimens led to now settled disputes about their species identity (Matschie 1893; Groves 1972). Images from Shaw (1792) and Audebert (1797) in public domain, made available by the Biodiversity Heritage Library

The first author who referenced the *Wou-wou* in connection with an available binomial name was the German scholar Anton August Heinrich Lichtenstein in his 1791 dissertation (year of publication verified e.g., by Brockhaus (1825)), several years before Audebert’s widely recognized treatise. His work was titled “*Commentatio philologica de simiarum quotquot veteribus innotuerunt formis earumque nominibus pro specimine methodi qua historia naturalis veterum ad systema naturae linnaeanum exigenda atque adornanda*” (when cited often abbreviated as “*De simiis veterum”*), and was released by the publishing house of Benjamin Gottlob Hoffmann. Therein, Lichtenstein (1791) reviewed references to primates in ancient Greek and Roman literature and tried to link them to the scientifically recognized species of his time. He eventually presented a synonymy of primate names, listing the species he deemed valid in compliance with the Linnean system, applying appropriate binomina. He distinguished the Javan gibbon from both the orangutan and the white-handed gibbon and named it *Simia Nanodes*, dwarf ape, etymologically inspired by a section in Aristotle’s *De partibus animalium* on “dwarves”. Although he concluded that ancient European scholars probably had knowledge of the white-handed gibbon (he interpreted the onocentaur beast from Claudius Aelianus’ *De natura animalium* as a transfigured lar gibbon), he doubted that they knew about the existence of the Javanese species. Nowadays, it is assumed that ancient Western authors did not have any notion of gibbons in general (Groves 2008).

Lichtenstein (1791) added the following brief description to his *Simia Nanodes*:

*Ecaudis, natibus calvis, brachiis longitudine corporis, capite obovato, facie nigra triangulari, serie pilorum ex albo cinereorum circumdata. *[Tailless, naked buttocks (referring to ischial callosities), arms as long as body, head obovate, face black and of triangular shape, framed by whitish-gray hair.]

Still, he pointed at the possibility that Javan and lar gibbon might at some point be identified as representatives of one morphologically variable species. The description was not accompanied by an illustration. As a locality for *Simia Nanodes* he provided:

*Habitat in India, praefertim in Java.* [Habitat in India (meaning South East Asia), preferably Java].

He further stated that “*wouwou”* and “*wauwau”* (the latter being the Germanized spelling of the former) are non-scientific terms referring to this species and cites a German translation (Von Wurmb 1783) of the treatise by Van Iperen and Schouwman (1780) as the principal source of information for his description. All that makes the identification of *Simia Nanodes* as the Javan gibbon unequivocal. In consequence, *Simia Nanodes* Lichtenstein, 1791 is a senior synonym of *Simia Moloch* Audebert, 1797. However, this case represents a reversal of precedence as defined in Article 23.9 of the International Commission on Zoological Nomenclature, preventing the older name to be used (ICZN 1999). To my best knowledge, Lichtenstein’s name has not been referenced to as valid in the scientific literature after 1899 (Article 23.9.1.1), while the one of Audebert has been universally accepted as such for decades (Article 23.9.1.2; see electronic supplement). Therefore, in compliance with the code, prevailing usage of *Hylobates moloch* (Audebert, 1797) must be maintained and *Simia Moloch* Audebert, 1797 is defined as a *nomen protectum* for the Javan gibbon. The senior synonym *Simia Nanodes* Lichtenstein, 1791 is herein declared a *nomen oblitum.*

Lichtenstein was a philologist interested in the conceptional history of primatology rather than a zoologist (despite being well versed in natural history), which could be the reason why his naming of the Javan gibbon remained undetected by the contemporary authorities on primates. It was, however, cited and in parts commented on by several German-speaking authors, most noteworthy Ludwig (1796), who incorrectly deemed one of the gibbons described by Buffon (1766) to be a member of the Javanese species. Nevertheless, just a few years after its publication, Lichtenstein’s name started to vanish from the literature. It was possibly last mentioned by Lesson (1840), who erroneously included *Sinia nanodes* [sic] in his synonymy of *Hylobates variegatus* (= *Hylobates lar* (Linnaeus, 1771)).

Unaffected by the reversal of precedence, the name-bearing type of the Javan gibbon remains a mounted specimen preserved at the Muséum National d’Histoire Naturelle in Paris (specimen number: MNHN-ZM-2005-970; photograph in Hendriksen (2019)). It was prominently illustrated alongside Audebert's (1797) description (Fig. 1c), which also indicates one non-depicted syntype, and was designated as a lectotype by Rode (1938) (he referred to it as a holotype, which is an invalid assignment according to Article 73.1.3 (ICZN 1999) since it has not been designated as such by the species’ describer). Originally, the lectotype specimen derives from the Dutch Stadholder’s collection that was curated by Arnout Vosmaer (Rode 1938; Hendriksen 2019). The latter is also known to have requested gibbons from the Royal Batavian Society for the Stadholder’s menagerie. However, all animals sent to him from Java apparently did not survive the passage (Van Iperen and Schouwman 1780). Whether the lectotype specimen was initially intended to be presented alive at the Dutch menagerie remains obscure. It eventually arrived in Paris following the seizure of the Stadholder’s collection by French authorities in 1795, shortly after the defeat of the Dutch republic in the Coalition wars (Lipkowitz 2014; Hendriksen 2019).

Below, I provide an updated synonymy of *Hylobates moloch* (Audebert, 1797) following stylistic recommendations by Gardner and Hayssen (2004). Lichtenstein’s *Simia Nanodes* is a further addition to the already complex nomenclatural history of the Javan gibbon, which was analyzed by Groves (1971, 1972) and Kappeler (1981). The now established name combination *Hylobates moloch* was first used by Frechkop (1934), a few years after it had been suggested as the Javan gibbon’s valid name by Cabrera (1930). It eventually gained more prominence after being employed by Chasen (1940) in his *Handlist of Malaysian Mammals*. However, Chasen (1940)*,* in contrast to Cabrera (1930) and Frechkop (1934)*,* subspecifically included Bornean (now commonly referred to as *H. muelleri* Martin, 1841 and *H. albibarbis* Lyon, 1911) as well as Javan gibbons into *H. moloch*, the taxonomic histories of which had long been intertwined (reviewed by Groves 1971). Only in the late 1970s and 1980s was this name increasingly used to refer to the Javan gibbon exclusively as a full species (Marshall and Marshall 1976; Chivers and Gittins 1978; Marshall and Sugardjito 1986). Before that, Javan gibbons were also frequently viewed as a subspecies of *Hylobates lar* (e.g., Groves 1972). Potential subspecific differentiation within the Javan gibbon and its nomenclatural implications were discussed by Dallmann and Geissmann (2009) but are not considered in the synonymy. Names designated to other gibbon species that were at times associated with *H. moloch*, as well as subgeneric assignments have been ignored.

## *Hylobates moloch* (Audebert, 1797)

*Simia Nanodes* Lichtenstein, 1791:31. Type locality “India praefertim in Java”. *Nomen oblitum*.

*Simia Moloch* Audebert, 1797: plate II. Type locality “Il habite les Moluques”. Restricted to “Tjianten, Mt. Salak, ca. 1100 m” by Sody, 1949:121). *Nomen protectum*.

*Simia cinerea* Cuvier, 1798:96 Type locality “De Batavia”. Preoccupied by *Simia cinerea* Kerr, 1792 (= *Mandrillus leucophaeus*).

*Simia Leucisca* Schreber, 1799: plate III B (actual publication year unknown, dated according to Sherborn (1892)). Type locality unknown. No description but artistic depiction.

*Pit.*[*Pithecus*] *cinereus*: Latreille, 1804:277. New name combination.

? *Simia hirsuta* Forster, mentioned in Sonnerat, 1806:81. Type locality unknown. *Nomen nudum*. (This citation derives from a reissue, the first edition of which is unavailable to the author.)

*Pithecus leuciscus*: Geoffroy Saint-Hilaire, 1812:89. New name combination.

*S.*[*Satyrus*] *Leuciscus*: Oken, 1816:1226. New name combination.

*Hylobates leuciscus*: Kuhl, 1820:6. New name combination. Not *Hylobates leuciscus* Matschie, 1893: 62 (= *Hylobates muelleri abbotti*).

*Cheiron Leuciscus*: Burnett, 1829:307. New name combination.

*H.[Hybolates] Leusiscus* Geoffroy Saint-Hilaire, 1834:34. Incorrect subsequent spelling of *Hylobates leuciscus* Kuhl, 1820.

*H.*[*Hylobates*] *leucurus* Gray, 1861:136. Incorrect subsequent spelling of *Hylobates leuciscus* Kuhl, 1820.

*Hylobates javanicus* Matschie, 1893:62. Type locality “Java”.

*Hylobates lar leuciscus*: Pocock, 1927:727. New name combination.

*Hylobates cinereus cinereus*: Kloss, 1929:119. New name combination.

*Hylobates moloch*: Frechkop, 1934:23. First use of current name combination (already alluded to but not written out in Cabrera, 1930).

*Hylobates moloch moloch*: Chasen, 1940:64. New name combination.

*Hylobates lar moloch*: Sody, 1949:121. New name combination.

*Hylobates lar pongoalsoni* Sody, 1949:123 Type locality ”Kali Kidang, Mount Slamat, C[entral]. Java, 800 m”. At times considered a subspecies (*H. moloch pongoalsoni*) (but see Dallmann and Geissmann (2009)).

## Electronic supplementary material

Below is the link to the electronic supplementary material.

Supplementary file1 (DOCX 15 kb)
