# Supplementary material for: Reversal of precedence: The oldest available name of the Javan gibbon and a complete synonymy of the species
Source: Primates. 2020 Apr 24;61(4):557–61. doi: 10.1007/s10329-020-00822-5 (PMC7347681; doi:10.1007/s10329-020-00822-5)
Supplement: Supplementary file 1 — Supplementary file1 (DOCX 15 kb) [file 10329_2020_822_MOESM1_ESM.docx]

A selection of publications that cite the name *Hylobates moloch* as valid, in compliance with the requirements specified in Article 23.9.1.2 of the International Code of Zoological Nomenclature to declare it a *nomen protectum*, is listed below:

Andayani N, Brockelman, W, Geissmann T, Nijman V, Supriatna, J. (2008) *Hylobates moloch* . The IUCN Red List of Threatened Species 2008: e.T10550A3199941. https://dx.doi.org/10.2305/IUCN.UK.2008.RLTS.T10550A3199941.en

Andayani N, Morales JC, Forstner MR., Supriatna, J, Melnick DJ (2001) Genetic variability in mtDNA of the silvery gibbon: implications for the conservation of a critically endangered species. Conservation Biology 15:770-775.

Brandon-Jones D, Eudey AA, Geissmann T, Groves CP, Melnick DJ, Morales JC, ... Stewart CB (2004) Asian primate classification. International Journal of Primatology 25:97-164.

Burns BL, Dooley HM., Judge DS (2011). Social dynamics modify behavioural development in captive white-cheeked (*Nomascus leucogenys*) and silvery (*Hylobates moloch*) gibbons. Primates 52:271–277.

Chan YC, Roos C, Inoue-Murayama M., Inoue E, Shih CC, Pei KJC, Vigilant L (2010). Mitochondrial genome sequences effectively reveal the phylogeny of *Hylobates* gibbons. PloS One. 2010;5:e14419.

Chatterjee HJ (2006) Phylogeny and biogeography of gibbons: a dispersal-vicariance analysis. International Journal of Primatology 27:699-712.

Chivers DJ, Gittins SP (1978) Diagnostic features of gibbon species. International Zoo Yearbook 18:157-164.

Dallmann R., Geissmann T (2001) Individuality in the female songs of wild silvery gibbons (*Hylobates moloch*) on Java, Indonesia. Contributions to Zoology 70:41-50.

Dallmann R., Geissmann T (2009) Individual and geographical variability in the songs of wild silvery gibbons (*Hylobates moloch*) on Java, Indonesia. In Lappan SM & Whittacker D (eds.) The gibbons: New perspectives on small ape socioecology and population biology. Springer, New York :91-110

Geissmann T (2002). Taxonomy and evolution of gibbons. Evolutionary Anthropology 11:28-31.

Geissmann T. & Nijman V (2006) Calling in wild silvery gibbons (*Hylobates moloch*) in Java (Indonesia): behavior, phylogeny, and conservation. American Journal of Primatology 68:1-19.

Groves CP (2001) Primate Taxonomy, Smithsonian Institution Press, Washington, DC.

Haimoff EH, Chivers DJ, Gittins SP, Whitten T (1982) A phylogeny of gibbons (*Hylobates* spp.) based on morphological and behavioural characters. Folia Primatologica 39:213-237.

Ham S, Lappan S, Hedwig D, Choe JC (2017). Female songs of the nonduetting Javan Gibbons (*Hylobates moloch*) function for territorial defense. International Journal of Primatology 38:533-552.

Hodgkiss S, Thetford E, Waitt CD, Nijman V (2010). Female reproductive parameters in the Javan gibbon (*Hylobates moloch*). Zoo Biology 29:449-456.

Kheng V, Zichello JM., Lumbantobing DN, Lawalata SZ, Andayani, N, Melnick DJ (2018) Phylogeography, population structure, and conservation of the Javan gibbon (*Hylobates moloch*). International Journal of Primatology 39:5-26.

Marshall JT, Sugardjito J (1986) Gibbon systematics. In Swindler DR & Erwin J (eds.) Comparative Primate Biology, Vol. 1: Systematics, Evolution, and Anatomy :137–185. Alan R. Liss, New York.

Mittermeier RA, Wilson DE, Rylands AB (eds.) (2013) Handbook of the mammals of the world: Primates. Lynx Edicions.

Mootnick AR (2006). Gibbon (Hylobatidae) species identification recommended for rescue or breeding centers. Primate Conservation 2006: 103-138.

Roos C (2016) Phylogeny and classification of gibbons (Hylobatidae). In Reichard UH, Barelli C, Hirai H (eds.) Evolution of Gibbons and Siamang :151-165. Springer, New York.

Supriatna J (2006) Conservation programs for the endangered Javan gibbon (*Hylobates moloch*). Primate Conservation 2006:155-162.

Takacs Z, Morales JC, Geissmann T, Melnick DJ (2005) A complete species-level phylogeny of the Hylobatidae based on mitochondrial ND3–ND4 gene sequences. Molecular Phylogenetics and Evolution 36:456-467.

Whittaker DJ, Morales JC, Melnick DJ (2007) Resolution of the *Hylobates* phylogeny: congruence of mitochondrial D-loop sequences with molecular, behavioral, and morphological data sets. Molecular Phylogenetics and Evolution 45:620-628.

Wilson DE, Reeder DM (eds.) (2005) Mammal species of the world: a taxonomic and geographic reference (Vol. 1). Johns Hopkins University Press, Baltimore.

Yi Y, Kim Y, Hikmat A, Choe JC (2020) Information transfer through food from parents to offspring in wild Javan gibbons. Scientific Reports 10, 714.
